# Pneumococcal vaccine coverage among individuals aged 18 to 64 years old with underlying medical conditions in the UK: a retrospective database analysis

**DOI:** 10.1186/s12889-020-09613-5

**Published:** 2020-10-21

**Authors:** Ian Matthews, Xiaoyan Lu, Qian Xia, Wynona Black, Bayad Nozad

**Affiliations:** 1 Merck, Sharp & Dohme Ltd., Hertford Road, Hoddesdon, EN11 9BU UK; 2 MSD, Lyon, France; 3 Merck & Co., Inc., Kenilworth, NJ USA; 4 Imperial College London, London, UK

**Keywords:** Vaccination, Time to vaccination, Vaccination coverage, pneumococcal vaccination, at-risk conditions

## Abstract

**Background:**

In the UK certain groups with pre-disposing conditions are eligible for vaccination with the pneumococcal polysaccharide vaccine (PPV23). Uptake of the vaccine in these individuals has not been reported for 10 years. Hence this study investigated the rates of pneumococcal vaccination, the time to vaccination since diagnosis, and factors associated with vaccination in individuals aged 18–64 years with certain underlying medical conditions.

**Methods:**

A retrospective database analysis was conducted using the Clinical Practice Research Datalink (CPRD). Individuals aged 18 to 64 years who had a diagnosis for underlying medical conditions of interest at the index date (January 1, 2011 to December 31, 2015) were included in this study. Both underlying conditions and pneumococcal vaccination were identified using Read codes. A multivariable logistic regression model was used to identify factors associated with pneumococcal vaccination.

**Results:**

A total of 99,153 individuals with underlying medical conditions were included in this study. Within 1 year of follow-up, 13.6% had received pneumococcal vaccination. This figure rose to 32.0% after 4 years of follow-up. The mean time between diagnosis and vaccination was 148.7 days across the overall cohort. Based on multivariate analysis of results, individuals with chronic heart disease, chronic kidney disease, chronic liver disease, chronic respiratory disease or diabetes mellitus were significantly less likely (*P* < 0.0001) to be vaccinated than those with immunosuppression. Individuals were significantly more likely to receive a pneumococcal vaccination if they received an influenza vaccination in the first year of follow-up than those who did not (*P* < 0.001).

**Conclusions:**

Despite the Joint Committee on Vaccination and Immunisation (JCVI) recommendations for pneumococcal vaccination in clinical risk groups, rates of pneumococcal vaccination are suboptimal in the UK for individuals aged 18–64 with underlying medical conditions. Further emphasis should be made on the importance of increased pneumococcal vaccination coverage in the UK, given the increased risk of morbidity and mortality associated with indicative underlying medical conditions.

## Background

Pneumococcal disease, caused by the gram-positive bacterium *S. pneumoniae,* is a major cause of invasive pneumococcal disease (IPD) and non-invasive pneumococcal diseases such as community-acquired pneumonia (CAP) and acute otitis media [1, 2]. IPD is of significant public health concern due to high morbidity and mortality globally, especially in the very young, the elderly and individuals with underlying medical conditions [3]. Patients with chronic and immunocompromising conditions have been shown to have substantially increased rates and costs of treatment for IPD and all-cause pneumonia (ACP) when compared with those of healthy counterparts. In 2017, the European Centre for Disease Prevention and Control (ECDC) received 23,886 notifications from 29 countries, of which 6333 cases (26.5%) were reported by the United Kingdom (UK). Furthermore, an estimated 1548 deaths due to IPD occurred within the European Union (EU) in 2017, of which 1018 were reported by the UK [4]. However, a number of countries did not report epidemiological data, and deaths reported may therefore be an underestimation [4]. The addition of influenza vaccine prior to influenza season has been shown to reduce the risk of severe courses of pneumonia, resulting in improved long-term survival [5]. However, the extent to which influenza vaccination may affect the rate of pneumococcal vaccination is unknown. In the UK, total annual healthcare costs related to pneumococcal disease are estimated to be greater than £1 billion [6]. Hospitalisations are an important contributor to this cost, with an estimated cost per episode of £4865 (patients aged 65 years) [7].

Individuals with underlying medical conditions have been targeted for pneumococcal vaccination to reduce the burden of pneumococcal disease in many countries worldwide [1]. Pneumococcal vaccination has been recommended in the UK in those with various clinical conditions since 1992, though uptake has been low [8]. Clinical risk groups have been defined as those with the following conditions: asplenia, chronic heart disease, chronic kidney disease, chronic liver disease, chronic respiratory disease, diabetes, immunosuppression, and individuals with cerebrospinal fluid leaks [9]. Cochlear implant patients were also considered, but the numbers were too small to report.

Currently, two pneumococcal vaccines are licensed and available for use in the UK in children over 5 years of age or adults at increased risk: the Pneumococcal Conjugate Vaccine (PCV13) which protects against 13 pneumococcal serotypes, and the Pneumococcal Polysaccharide Vaccine (PPV23) which protects against 23 pneumococcal serotypes [9]. In the UK, pneumococcal vaccination is typically administered in general practice.

The national pneumococcal immunisation programme for the UK was introduced in 2003 and initially offered PPV23 vaccination to all adults aged 80 years and older. By 2005, the programme had been extended to offer vaccination to all adults aged 65 years and over who were not previously immunised. In 2009, assessment of PPV23 vaccine uptake in patients aged 2–64 years within clinical risk groups was added to the annual survey of vaccine uptake [3, 10].

Current real-world data on pneumococcal vaccination coverage rates (VCR) in individuals aged 18–64 years with underlying medical conditions in the UK is limited. A 2012 Department of Health (DoH) report described data collected on at-risk patients vaccinated any time up and until 31st March 2009 [10]. It was reported that VCRs in at-risk individuals aged 16–64 years were only 34.4% during this time period in the UK. However, since 2009 the DoH have reported only the annual VCR in adults aged 65 years and older. Therefore, there is currently no recent UK report of the VCR for individuals with underlying medical conditions.

## Methods

### Study aim, design and data source

The objective of this study was to examine the pneumococcal VCR, potential factors associated with pneumococcal vaccination uptake, and time since diagnosis to pneumococcal vaccination in individuals in the UK with underlying medical conditions.

A retrospective cohort study was conducted using anonymised Electronic Health Record (EHR) data from the CPRD, a database jointly sponsored by the Medicines and Healthcare products Regulatory Agency (MHRA) and the National Institute for Health Research (NIHR), as part of the DoH in the UK. The CPRD contains over 13 million records, of which 5 million are active, and is drawn from approximately 650 primary care practices in the UK. This database contains clinical and prescription data, which is a valuable source of information to inform policy makers by supporting real world public health research.

### Study variables

Underlying medical conditions of interest within this study included the following: asplenia, chronic heart disease, chronic liver disease, chronic kidney disease, chronic respiratory disease, diabetes, and immunosuppression. These variables were chosen based on the clinical risk groups recommended for pneumococcal vaccination within the Green Book [9], and data availability within the CPRD.

Underlying medical conditions were identified solely by Read code (a coded thesaurus of clinical terms used by primary care within the National Health Service in the UK). Persons without evidence of these underlying medical conditions were classified as healthy.

Pneumococcal vaccination was also identified by Read codes. The pneumococcal VCR was calculated for 1 year following the index date (defined as the date of the first diagnosis of any of the underlying medical conditions of interest between January 1, 2011 to December 31, 2015) and for the average follow-up period to examine whether pneumococcal vaccination had been given.

VCR was defined as:

$$ \frac{\left( Number\ of\ individuals\  who\  received\ pneumococcal\ vaccine\right)\ast 100}{\left( Number\ of\ individuals\  who\  are\  newly\ diagnosed\ with\kern0.5em underlying\ medical\ conditions\ of\ interest\right)} $$

Univariate and full Cox analysis were also conducted to examine the factors associated with vaccine uptake.

### Study sample and follow-up period

**Fig. 1:**
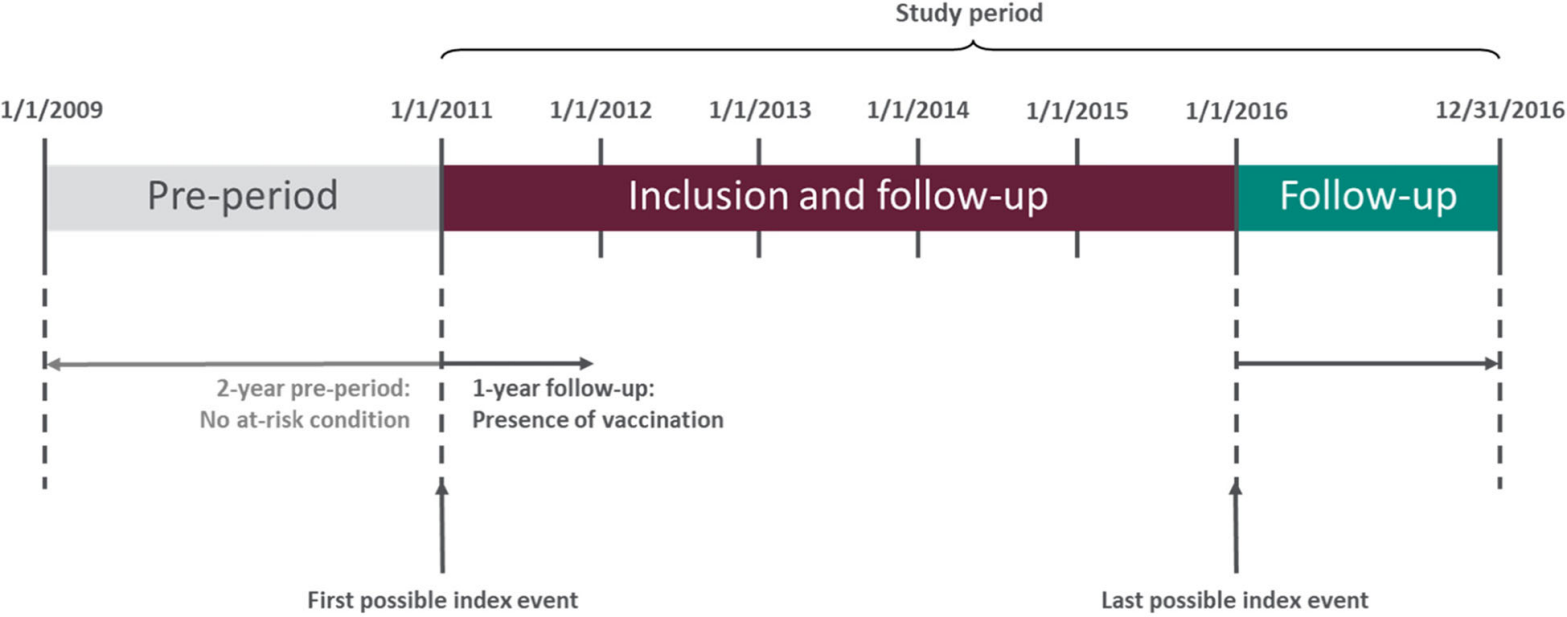
Study Design

Individuals who were at least 18 years of age and before their 65th birthday with a clinical diagnosis for one of the conditions of interest at the index date (January 1, 2011 to December 31, 2015) were included. Individuals were required to have had continuous enrolment in the database for at least 2 years before the index date to ensure no presence of any underlying medical condition of interest, and for 1 year following the index date (see Fig. 1). Individuals who had any underlying medical conditions of interest within 2 years (730 days) prior to the index date or a record of vaccination were excluded.

## Results

### Study cohort

**Fig. 2:**
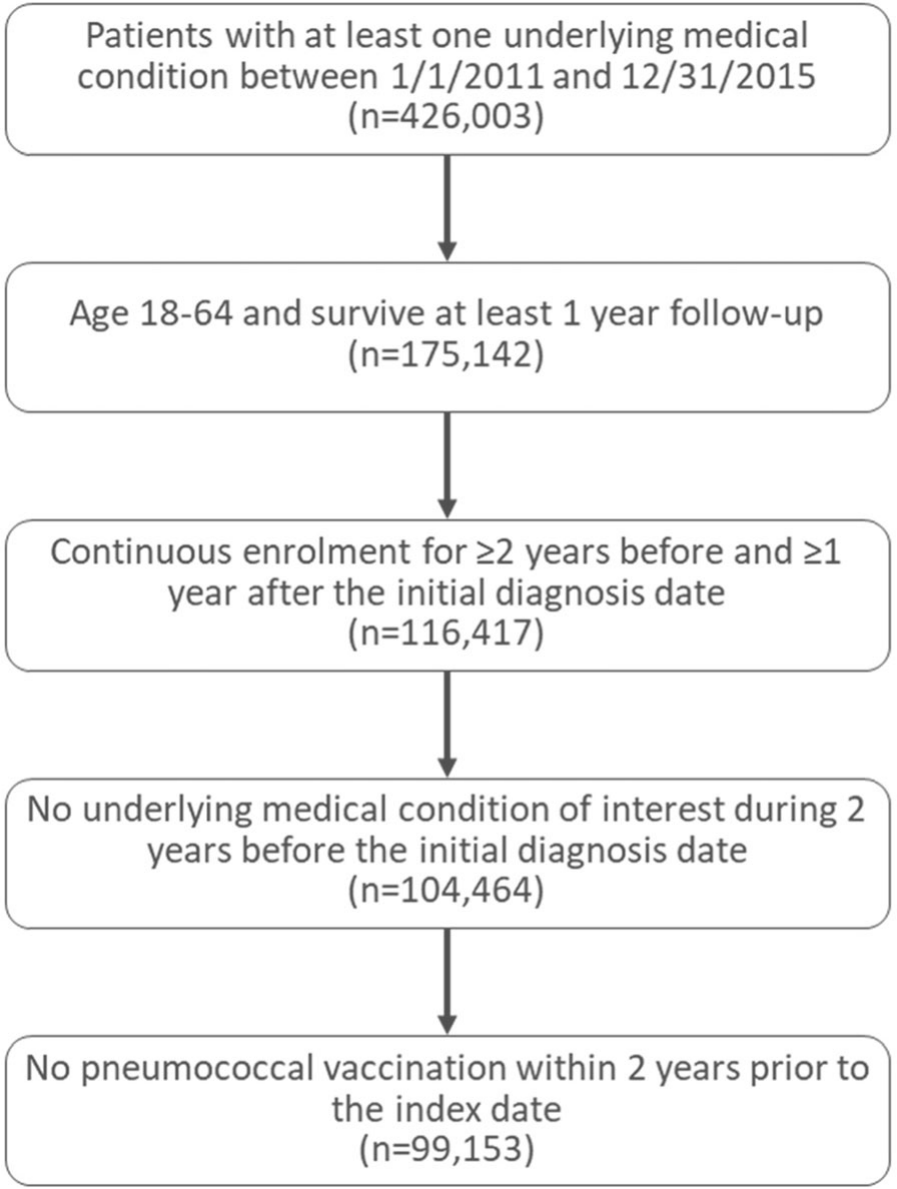
Flow Chart Of Study Sample Identification

Cohort construction is reported in Fig. 2. A total of 426,003 individuals with at least one underlying medical condition between 1/1/2011 and 12/31/2015 were identified, of which 99,153 were eligible for inclusion in the study.

Full demographics are reported in Table 1. A total of 99,153 individuals aged 18–64 years between January 1, 2011 to December 31, 2015 with at least one underlying medical condition of interest were identified. The mean age of individuals was 51.8 years (standard deviation [SD] = 10.1 years) and the majority of individuals were aged 50–64 years (66.5%). Males comprised 56.6% of the sample. Of the regions studied, the largest number of individuals were from Scotland (15.4%). The most common underlying medical condition was diabetes mellitus (40.4%), followed by chronic respiratory disease (22.6%). Nearly two thirds of patients received influenza vaccine in the first year of follow-up post-index date (64.3%).

**Table 1:** Characteristics And Pneumococcal VCR (%) Of Individuals Aged 18–64 Years With Newly Diagnosed Underlying Medical Conditions (CPRD; 2011–2015)

	Total	Pneumococcal vaccine in the 1-year follow-up		***P***-value	VCR
	N (%)	N (%)			(%)
		Yes	No		
Total	99,153 (100)	13,518 (100)	85,635 (100)		13.6
**Age**				<.0001	
Mean years (SD)	51.8 (10.1)	52.8 (9.5)	51.7 (10.2)		
18–49	33,210 (33.5)	4143 (30.7)	29,067 (33.9)		12.5
50–64	65,943 (66.5)	9375 (69.4)	56,568 (66.1)		14.2
**Gender**^**a**^				0.0304	
Male	56,139 (56.6)	7770 (57.5)	48,369 (56.5)		13.8
Female	43,009 (43.4)	5748 (42.5)	37,261 (43.5)		13.4
**Region**				<.0001	
North East	1366 (1.4)	126 (0.9)	1240 (1.5)		9.2
North West	11,777 (11.9)	1727 (12.8)	10,050 (11.7)		14.7
Yorkshire and The Humber	1672 (1.7)	290 (2.2)	1382 (1.6)		17.3
East Midlands	520 (0.5)	75 (0.6)	445 (0.5)		14.4
West Midlands	8743 (8.8)	1363 (10.1)	7380 (8.6)		15.6
East of England	4959 (5.0)	712 (5.3)	4247 (5.0)		14.4
South West	7188 (7.2)	947 (7.0)	6241 (7.3)		13.2
South Central	9143 (9.2)	1249 (9.2)	7894 (9.2)		13.7
London	10,381 (10.5)	1295 (9.6)	9086 (10.6)		12.5
South East Coast	9210 (9.3)	1137 (8.4)	8073 (9.4)		12.3
Northern Ireland	4312 (4.3)	613 (4.5)	3699 (4.3)		14.2
Scotland	15,241 (15.4)	2676 (19.8)	12,565 (14.7)		17.6
Wales	14,641 (14.8)	1308 (9.7)	13,333 (15.6)		8.9
**Individuals with one underlying medical condition**^**b**^				<.0001	
Cerebrospinal fluid leaks	211 (0.2)	15 (0.1)	196 (0.2)		7.1
Chronic heart disease	18,103 (18.3)	2228 (16.5)	15,875 (18.5)		12.3
Chronic kidney disease	1871 (1.9)	162 (1.2)	1709 (2.0)		8.7
Chronic liver disease	2697 (2.7)	199 (1.5)	2498 (2.9)		7.4
Chronic respiratory disease	22,367 (22.6)	3110 (23.0)	19,257 (22.5)		13.9
Diabetes mellitus	40,035 (40.4)	5775 (42.7)	34,260 (40.0)		14.4
Immunosuppression	10,125 (10.2)	1340 (9.9)	8785 (10.3)		13.2
**Influenza vaccine use in the first year of follow-up**				<.0001	
No	35,405 (35.7)	887 (6.6)	34,518 (40.3)		2.5
Yes	63,748 (64.3)	12,631 (93.4)	51,117 (59.7)		19.8

*CPRD* Clinical Practice Research Datalink, *SD* Standard deviation, *VCR* Vaccination coverage rate.

^a^Five patients did not provide a male/female code within the suggested data field, and have therefore not had gender recorded

^b^Patients with multiple chronic conditions (*n* = 3728) or cochlear implants (*n* = 16) were also recorded, but were excluded from final analysis

### Vaccination coverage rates during first year of diagnosis

**Fig. 3:**
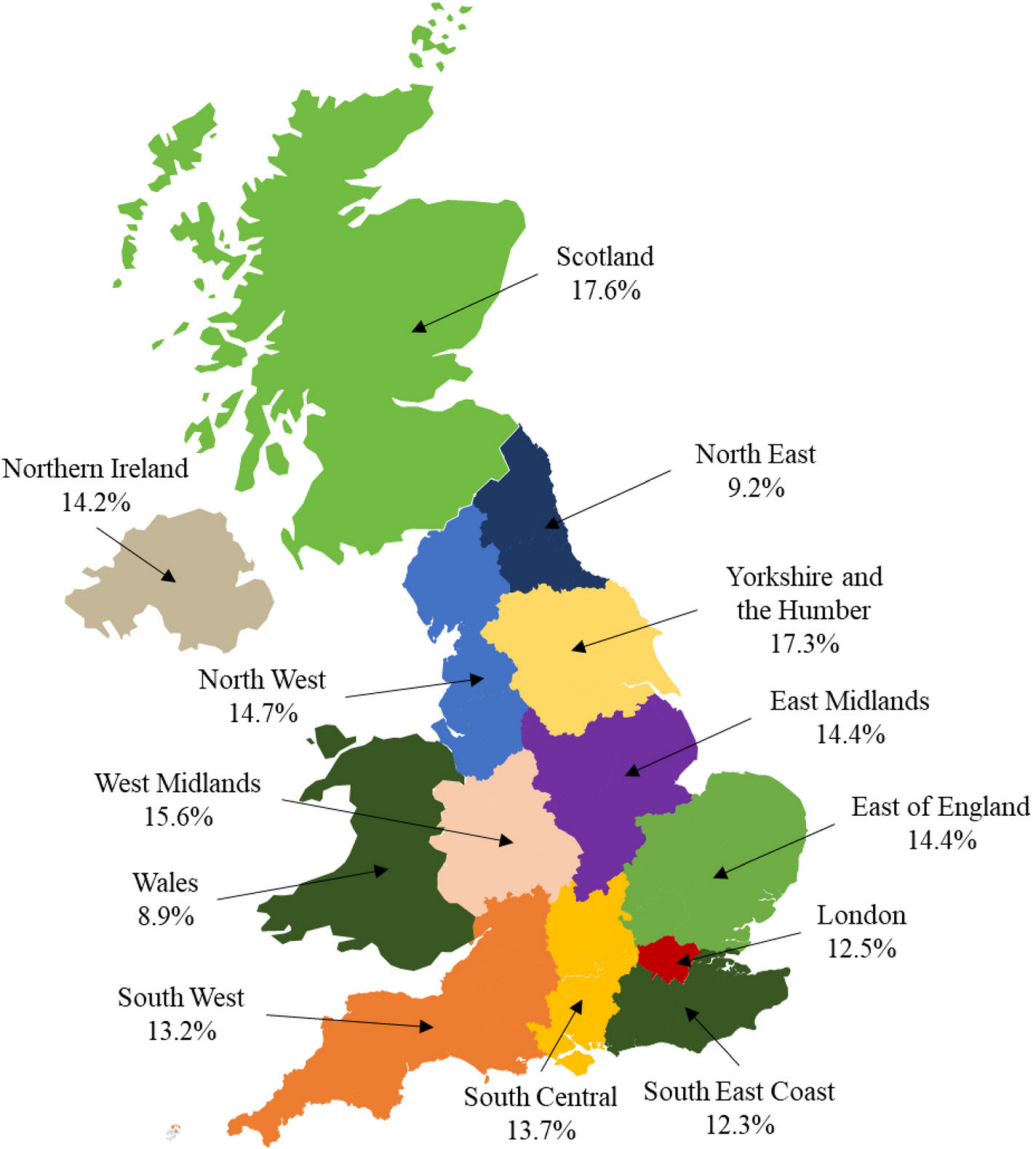
Vaccine Coverage Rates (%) By UK Region (CPRD; 2011–2015). CPRD: Clinical Practice Research Datalink; UK: United Kingdom. This figure was created using data from the CPRD and a Microsoft PowerPoint template from yourfreetemplates.com

Table 1 presents the factors associated with vaccination coverage among individuals aged 18–64 years with underlying medical conditions over a one-year period from index date. Of the overall cohort, 13.6% had received pneumococcal vaccination, whilst 86.4% were unvaccinated. Figure 3 presents the VCR across the UK. Those from Scotland had higher rates of vaccination (17.6%) than those from Northern Ireland (14.2%) or Wales (8.9%). However, there was substantial variation in vaccination rates across regions within England. The highest VCR were seen in Yorkshire and the Humber (17.3%), followed by the West Midlands (15.6%), whilst the lowest rates were seen in the North East (9.2%). Across all regions, individuals with diabetes mellitus had the highest rates of pneumococcal vaccination (14.4%), followed by chronic respiratory disease (13.9%) and immunosuppression (13.2%). Individuals with cerebrospinal fluid leaks (7.1%) had the lowest rates of vaccination coverage.

Those who received influenza vaccine in the first year of follow-up post index date had higher rates of pneumococcal vaccination coverage (19.8%) than those who had not (2.5%).

### Vaccination coverage rates over a four-year period

**Fig. 4:**
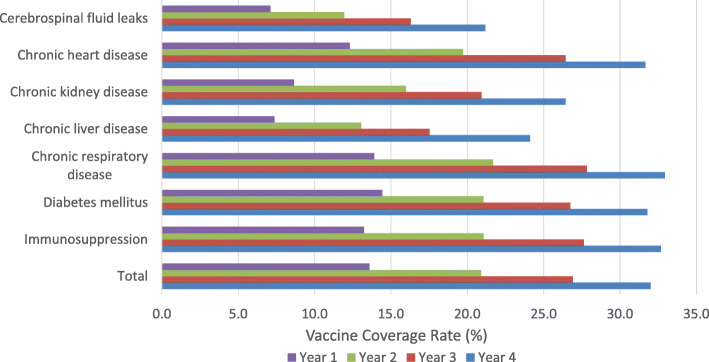
Vaccine Coverage Rates (%) By Year Following Diagnosis (CPRD; 2011–2015). CPRD: Clinical Practice Research Datalink

Figure 4 presents the VCR among individuals aged 18–64 years with underlying medical conditions over a four-year period. Of the overall cohort, 13.6% had received pneumococcal vaccination after 1 year, rising to 32.0% by year four. After 4 years of follow-up, individuals with chronic respiratory disease (32.9%) had the highest rates of pneumococcal vaccination followed by those with immunosuppression (32.7%) and diabetes mellitus (31.8%). Individuals with cerebrospinal fluid leaks (21.2%) had the lowest rates of vaccination coverage. The highest rate of change over 4 years was seen in individuals with chronic liver disease (226% increase) and chronic kidney disease (205% increase), whilst the lowest rate of change was seen in individuals with diabetes mellitus (120%).

### Time to vaccination

Table 2 presents the time between initial diagnosis to receipt of vaccination in individuals aged 18–64 years with underlying medical conditions across the duration of the study. The mean time between diagnosis and vaccination was 148.7 days (approximately 5 months) across the overall cohort. Among individuals with only one medical condition, the mean interval between diagnosis and vaccination was highest in those with chronic liver disease (176.3 days) followed by chronic heart disease (170.7 days) and chronic kidney disease (168.7 days). The mean interval between diagnosis and vaccination was lowest in those with immunosuppression (130.6 days).

**Table 2:** Average Interval From Initial Diagnosis To Pneumococcal Vaccination In Individuals With Only One Underlying Medical Condition^a^ (CPRD; 2011–2015)

Medical condition	N	Days, median	Days, mean (SD)
Total	13,518	133	148.7 (107.2)
Cerebrospinal fluid leaks	15	146	135.5 (118.1)
Chronic heart disease	2228	164	170.7 (103.9)
Chronic kidney disease	162	167	168.7 (105.2)
Chronic liver disease	199	171	176.3 (99.8)
Chronic respiratory disease	3110	135	150.4 (108.1)
Diabetes mellitus	5775	121	140.3 (106.3)
Immunosuppression	1340	98	130.6 (108.0)

^a^Patients with multiple chronic conditions (*n* = 3728) or cochlear implants (*n* = 16) were also recorded, but were excluded from final analysis

*CPRD* Clinical Practice Research Datalink, *SD* Standard deviation.

### Factors associated with pneumococcal vaccination uptake

Table 3 presents the factors associated with pneumococcal vaccination uptake for those with at least 1 year of follow up.

**Table 3:** Factors Associated With Pneumococcal VCR (CPRD; 2011–2015)

	Univariate logistic regression		Full Cox regression	
Variables	HR (95% CI)	P-value	HR (95% CI)	***P***-value
**Age**				
18–49	Reference group		Reference group	
50–64	1.14 (1.10, 1.19)	*P* < 0.0001	0.96 (0.93, 1.00)	*P* = 0.051
**Gender**				
Male	Reference group		Reference group	
Female	0.96 (0.93, 1.00)	*P* = 0.033	0.91 (0.88, 0.95)	*P* < 0.0001
**Region**				
North East	Reference group			
North West	1.64 (1.37, 1.97)	*P* < 0.0001	1.52 (1.27, 1.82)	*P* < 0.0001
Yorkshire and the Humber	1.96 (1.59, 2.42)	*P* < 0.0001	1.98 (1.60, 2.44)	*P* < 0.0001
East Midlands	1.60 (1.20, 2.13)	*P* = 0.001	1.49 (1.12, 1.99)	*P* = 0.006
West Midlands	1.75 (1.46, 2.10)	*P* < 0.0001	1.66 (1.39, 2.00)	*P* < 0.0001
East of England	1.60 (1.32, 1.93)	*P* < 0.0001	1.57 (1.30, 1.90)	*P* < 0.0001
South West	1.46 (1.21, 1.75)	*P* < 0.0001	1.45 (1.21, 1.75)	*P* < 0.0001
South Central	1.51 (1.25, 1.81)	*P* < 0.0001	1.48 (1.23, 1.77)	*P* < 0.0001
London	1.37 (1.14, 1.65)	*P* = 0.0001	1.30 (1.08, 1.56)	*P* = 0.005
South East Coast	1.36 (1.13, 1.64)	*P* = 0.001	1.34 (1.12, 1.61)	*P* = 0.002
Northern Ireland	1.57 (1.30, 1.90)	*P* < 0.0001	1.38 (1.14, 1.68)	*P* = 0.001
Scotland	1.98 (1.65, 2.37)	*P* < 0.0001	1.92 (1.61, 2.30)	*P* < 0.0001
Wales	0.96 (0.80, 1.16)	*P* = 0.677	1.02 (0.85, 1.22)	*P* = 0.870
**Individuals with only one underlying medical condition**				
Immunosuppression	Reference group		Reference group	
Cerebrospinal fluid leaks	0.52 (0.31, 0.86)	*P* = 0.01	0.77 (0.47, 1.29)	*P* = 0.322
Chronic heart disease	0.91 (0.85, 0.97)	*P* = 0.006	0.71 (0.66, 0.76)	*P* < 0.0001
Chronic kidney disease	0.63 (0.54, 0.74)	*P* < 0.0001	0.55 (0.46, 0.64)	*P* < 0.0001
Chronic liver disease	0.53 (0.46, 0.62)	*P* < 0.0001	0.55 (0.47, 0.64)	*P* < 0.0001
Chronic respiratory disease	1.05 (0.98, 1.11)	*P* = 0.181	0.86 (0.80, 0.91)	*P* < 0.0001
Diabetes mellitus	1.09 (1.03, 1.16)	*P* = 0.004	0.82 (0.77, 0.88)	*P* < 0.0001
**Influenza vaccine use in the first year of follow-up**				
No	Reference group		Reference group	
Yes	8.68 (8.11, 9.29)	*P* < 0.0001	8.78 (8.19, 9.40)	*P* < 0.0001

*CI* Confidence interval, *CPRD* Clinical Practice Research Datalink, *HR* Hazard ratio, *VCR* Vaccination coverage rate

Based on the univariate analysis of results, older age groups (aged 50–64 years) were 14% more likely to receive pneumococcal vaccination than those aged 18–49 (*P* < 0.0001). Women were 4% less likely to receive vaccination than men (*P* = 0.033). Further, all regions except Wales were significantly more likely to be vaccinated compared to individuals from the North East (All: *P* < 0.01).

Also, it was found that patients with cerebrospinal fluid leaks, chronic heart disease, chronic kidney disease, and chronic liver disease were significantly less likely to be vaccinated than patients with immunosuppression (All: *P* ≤ 0.01). Conversely, patients with diabetes mellitus were significantly more likely to be vaccinated than patients with immunosuppression (*P* = 0.004). Patients with chronic respiratory disease did not have a significant association with VCR compared to the reference group (*P* = 0.181).

In multivariate analysis, there was a non-significant trend towards older age groups being less likely to receive vaccination (HR = 0.96, *P* = 0.051). Women were still significantly less likely to receive pneumococcal vaccination than men (HR = 0.91, *P* = < 0.0001).

Similar findings to the univariate analysis were found in terms of vaccine uptake in different regions in the multivariate analysis.

Furthermore, the multivariate analysis showed that patients with chronic heart disease, chronic kidney disease, chronic liver disease, chronic respiratory disease, and diabetes mellitus were from 14 to 45% less likely to be vaccinated than patients with immunosuppression (All: *P* < 0.0001). There was no significant difference between the likelihood of vaccination for patients with cerebrospinal fluid leaks and patients with immunosuppression (*P* = 0.322). Following the adjustment, patients with chronic respiratory disease and diabetes mellitus had significantly lower VCR compared to immunosuppressed patients.

Finally, in both analyses conducted, individuals were over eight times more likely to receive a pneumococcal vaccination if they received an influenza vaccination in the first year of follow-up post index date than those who did not (Both: *P* < 0.0001).

## Discussion

Our findings provide insight into pneumococcal vaccination patterns in individuals aged 18–64 years with underlying medical conditions in the UK. Despite the national recommendation for pneumococcal vaccination in clinical risk groups, only 13.6% of patients newly diagnosed with underlying medical conditions received a pneumococcal vaccination after 1 year of follow-up. After 4 years of follow-up, this rose to 32.0%. These low vaccination rates are disappointing, given the increased risk of pneumococcal disease in individuals with underlying medical conditions, and indicate that patients would benefit from measures to increase pneumococcal VCR within the UK. Consistent with previous research on factors associated with vaccine uptake, gender was significantly associated with pneumococcal vaccination in the univariate and multivariate analysis, respectively. There was also a non-significant trend that suggested age was associated with vaccine uptake. The improved rate of vaccination amongst those aged 50–64 may be due to increased physician contact, which has previously been associated with a significant increase in vaccination coverage [11]. A further factor may be that adults aged 50–64 are more likely to have comorbidities than those aged 18–49, and may have greater opportunity to be vaccinated. Similarly, it has been widely documented that rates of vaccination are greater for males than females [12], despite women having a higher prevalence of physician visits overall [13].

Substantial variation in vaccination rates was noted across regions of the UK and within England, with all regions except Wales having significantly better vaccination rates than the North East of England. This is perhaps due to the larger health inequalities experienced by patients in the North East. For example, a recent analysis found that primary care is underfunded in the North East, relative to the level of morbidity [14], which may result in lower vaccination rates. However, it should be noted that large differences in patient numbers were reported across regions of England, whilst variations in data collection and reporting procedures may also contribute to the disparity of results. Further investigation is required to identify the reasons for age-related, gender, and regional disparities in vaccination rates across the UK.

Current knowledge on pneumococcal vaccination coverage in individuals aged 18–64 years with underlying medical conditions is limited. A previously conducted DoH survey found that pneumococcal vaccine uptake reached 34.4% in individuals aged 16–64 years who were considered “at-risk” [10]. This correlates with the findings of this study, in which pneumococcal vaccine uptake reached 32.0% in individuals aged 18–64 years with underlying medical conditions. The slight difference in vaccination rates may be accounted for by the difference in study lengths, as this study was limited to a four-year period following diagnosis, whereas the DoH study accounted for patients receiving PPV vaccine any time until 2009. However, the results presented indicate that VCRs remain low within the population of interest, and further efforts should be made to increase the vaccination coverage within the UK.

After 1 year of follow-up, VCR in patients with one underlying medical condition were highest in those with diabetes mellitus (14.4%), and lowest in those with cerebrospinal fluid leaks (7.1%). After 4 years of follow-up, VCR rose in these patients to 31.8 and 21.2% respectively. Although there is an interval of nearly 10 years, these rates are comparable to those produced by the DoH where vaccination rates were lowest in those with cerebrospinal fluid leaks (16.3%) [10]. Given that age ranges and indications examined were similar between studies, the VCR discrepancy may in part be due to the low numbers of individuals diagnosed with cerebrospinal fluid leaks within the study period, which may result in inaccurate estimates of VCR rates within this population.

A 2019 study has also been conducted by Public Health England in at-risk patients aged 65 years and older, who were diagnosed and eligible for pneumococcal vaccination between April 2017 and March 2019. Within this report, individuals with cochlear implants had the highest rates of pneumococcal vaccination (52.2%), followed by diabetes mellitus (19.7%) and asplenia (17.8%). Individuals with cerebrospinal fluid leaks (6.5%) had the lowest rates of vaccination coverage [15]. The results presented by Public Health England mirror those found within this study, despite the different age ranges studied. However, it should also be noted that the number of patients diagnosed with each condition were not reported, and therefore direct comparisons cannot be applied.

Finally, individuals who received influenza vaccine in the first year of follow-up post index date were significantly more likely to receive pneumococcal vaccination than those who did not (*P* < 0.0001). Individuals with underlying medical conditions are recommended to receive influenza vaccination. The influenza season is annual, with focused national and local programmes to increase vaccine uptake – unlike pneumococcal vaccination. Therefore, the seasonal influenza programme offers an opportunity to provide pneumococcal vaccination alongside the influenza vaccine to unvaccinated individuals in risk groups. In 2014, NHS England introduced Enhanced Services specification to aid general practitioner (GP) practices in delivering seasonal influenza and pneumococcal vaccination programmes concurrently. As pneumococcal infection is a recognised complication of influenza, providing the two vaccines together early in the season will increase the level of protection to individuals with underlying conditions [16], and may result in improved long-term survival [5], while reducing appointment and administration costs.

### Limitations

As a primary care database, CPRD has a number of limitations in terms of recording medical conditions. Immunosuppressive conditions (such as human immunodeficiency virus [HIV]) are widely underreported in the primary care setting due to a number of reasons. These include patient confidentiality, coding being unable to allow detailed analysis of transplants leading to immunodeficiency, and certain immunosuppressive therapies which may not be completely captured due to their non-primary care usage. Finally, vaccinations received before or after the study period were not captured, as laid out within the methodology of the study. Consequently, CRPD data may not capture the true vaccination rates in patients with IPD. Whilst this study is subject to limitations in collecting data from primary care sources, including variations in data reporting and collection across GP catchment areas, this is an important analysis of a robust data source that reflects real world practice to measure VCR within this population.

Additionally, the authors acknowledge a limitation in the chosen statistical analysis using the multivariate method. It would be recommended that this multivariate analysis be investigated further in future studies.

## Conclusion

Despite UK recommendations for pneumococcal vaccination in clinical risk groups, rates of pneumococcal vaccination may be suboptimal in UK individuals aged 18–64 with underlying medical conditions, with only 13.6% of the overall cohort receiving vaccination within 1 year of index date, rising to 32.0% after 4 years of follow-up. Further emphasis should be made on the importance of increased pneumococcal vaccination coverage in the UK, given the increased risk of morbidity and mortality within individuals with underlying medical conditions. Improved messaging on the importance of vaccination is key to improving vaccination rates. The results of this study reveal the low rate to decision makers, thereby helping to stimulate the development of strategies for addressing barriers to pneumococcal vaccination.

## Data Availability

Source data for this study were obtained from the Clinical Practice Research Datalink (CPRD). These data sources are made available for scientific and medical research after submission of a study protocol to be reviewed and approved by the CPRD Independent Scientific Advisory Committee (ISAC). Owing to ethical restrictions, the data used in this analysis are not publicly available, in line with the data privacy rules set up by CPRD/ISAC. Data access queries can be directed to enquiries@cprd.com.

## Abbreviations

ACP: All cause pneumonia
CAP: Community acquired pneumonia
CI: Confidence interval
CPRD: Clinical Practice Research Datalink
DoH: Department of Health
ECDC: European Centre for Disease Prevention and Control
EHR: Electronic health record
GP: General practitioner
HIV: Human immunodeficiency virus
IPD: Invasive pneumococcal disease
JCVI: Joint Committee on Vaccination and Immunisation
MHRA: Medicines and Healthcare products Regulatory Agency
NIHR: National Institute for Health Research
PCV13: Pneumococcal conjugate vaccine
PPV23: Pneumococcal polysaccharide vaccine
SD: Standard deviation
UK: United Kingdom
VCR: Vaccination coverage rates
